# Characterization of Room and Elevated Temperature Mechanical Properties of P22/P91 Weld Metals Using Small Punch Tensile and Uniaxial Tensile Tests

**DOI:** 10.3390/ma19194085

**Published:** 2026-09-24

**Authors:** Shuo Zhang, Yifan Hu, Xiangfeng Zheng, Lei Zhao, Yue Zhao, Yunchen Xie, Shuyu Li, Yuehong Dong

**Affiliations:** 1 China Energy Science and Technology Research Institute Co., Ltd., Nanjing 210023, China; 20103428@ceic.com (Y.H.); 12054731@ceic.com (X.Z.); 20080597@ceic.com (Y.Z.); 12074463@ceic.com (Y.X.); 12036891@ceic.com (S.L.); 12067694@ceic.com (Y.D.); 2 CHN Energy Boiler & Pressure Vessel Inspection Co., Ltd., Beijing 102200, China; 3 School of Materials Science and Engineering, Tianjin University, Tianjin 300072, China; 4 National Key Laboratory of Low-Carbon Intelligent Coal-Fired Power Generation and Ultra-Clean Emission, Nanjing 210023, China

**Keywords:** P22, P91, weld metal, uniaxial tensile test, small punch tensile test

## Abstract

P22 and P91 heat-resistant steel weld metals (P22WM, P91WM) were tested by uniaxial tensile (UT) and small punch tensile (SPT) tests at room and service temperatures. Their high-temperature mechanical properties, fracture features and UT-SPT mechanical property correlation were systematically studied. P91WM exhibits approximately 43% higher yield strength at room temperature (RT) compared with P22WM. Nevertheless, P22WM retains around 24% more strength relative to its RT value under service temperature, showing better high-temperature stability. Multiple linear UT-SPT correlation models are established, where the Mao model achieves the highest prediction accuracy for yield strength, and the Altstadt model (ALT) exhibits superior ultimate tensile strength (UTS) prediction performance over the classical model (CLA). SPT is proven to efficiently characterize the mechanical properties of heat-resistant steel weld metals, supporting the in-service evaluation of high-temperature welded joints.

## 1. Introduction

High-temperature pressure-bearing pipelines and key components in high-end equipment such as ultra-supercritical (USC) thermal power units and petrochemical facilities are chronically exposed to harsh service environments coupled with high temperature, high pressure and combined loading. Such conditions place stringent demands on the high-temperature mechanical properties, creep resistance and oxidation resistance of structural materials. P22 (2.25Cr-1Mo) pearlitic–bainitic low-alloy heat-resistant steel and P91 (9%Cr) martensitic heat-resistant steel are widely employed as core structural materials for the aforementioned equipment, owing to their exceptional high-temperature stability and cost-effectiveness [1,2,3,4,5,6]. P22 steel possesses favorable cost performance and excellent intermediate-temperature mechanical properties, making it applicable to subcritical power units and conventional medium-temperature petrochemical components [7,8,9,10,11,12]. In contrast, P91 steel exhibits significantly enhanced high-temperature strength and creep resistance via alloy optimization and grain refinement strengthening [13], thereby satisfying the service requirements of high-parameter facilities, including USC power units [14,15,16].

Welding represents the core manufacturing and repair process for heat-resistant steel components. As the load-bearing core of welded joints, weld metal (WM) differs distinctly from the base metal in chemical composition, microstructure and mechanical properties. Consequently, it acts as the critical region prone to high-temperature failure in welded joints, which directly determines the operational safety and service life of in-service equipment [17,18,19,20,21].

The conventional uniaxial tensile (UT) test serves as the standard method for characterizing the high-temperature mechanical properties of metallic materials. Nevertheless, this method requires standard large-scale specimens, making it difficult to extract samples from narrow weld regions. It cannot achieve precise property characterization of micro-zones in welded joints, nor can it meet the requirements for micro-damage, in situ and localized performance evaluation of in-service components [22,23]. As a miniature specimen mechanical testing technique, the small punch tensile (SPT) test only requires tiny disc specimens with a diameter of 8–10 mm and a thickness of 0.5 mm. It enables the inverse derivation of key mechanical parameters such as yield strength, tensile strength and elongation from load–displacement curves. With prominent merits, including flexible sampling, minor destructive testing, localized property characterization and low cost, SPT has become an ideal technical approach for evaluating the high-temperature properties of heat-resistant steel welded joints [24,25,26,27,28].

To date, abundant small punch tensile (SPT) studies have been carried out on P22 and P91 heat-resistant steels, with the vast majority targeting their base metals (BM). Through these efforts, a series of classic UT-SPT correlation models—such as the Mao, CEN, and *t*_0_/10 model, the classical model (CLA) [29], and Altstadt (ALT) [30] models—have been well established and validated, enabling reliable conversion of SPT characteristic parameters into uniaxial tensile (UT) properties, including yield strength (YS) and ultimate tensile strength (UTS), for P22 and P91 base materials. For instance, García et al. [31] proposed the CLA model based on tests on several steels, including 2.25Cr-1Mo (P22) base metal, and Milička & Dobeš [32] established empirical relationships between SPT and conventional tensile results for P91 base metal at both room and elevated temperatures. More recently, Zhang et al. [33] validated multiple UT-SPT correlation models for both P22 and P91 base metals with high prediction accuracy. In contrast, systematic investigations focusing specifically on the weld metals (WM) of these steels remain remarkably limited. While a few studies have touched upon P91 weld metal incidentally [34], no dedicated UT-SPT correlation model has been reported that simultaneously fits both P22WM and P91WM fabricated under specified welding processes. Furthermore, a comprehensive verification of prediction accuracy and error distribution for relevant models on these weld metals is still lacking [35]. To the best of our knowledge, no UT-SPT correlation study has been reported for P22 weld metal at all, irrespective of the welding process. These critical gaps hinder the rapid and accurate evaluation of high-temperature mechanical properties for in-service welded joints in engineering applications [36,37].

In this work, P22WM and P91WM prepared by gas tungsten arc welding (GTAW) for root passes, shielded metal arc welding (SMAW) for filling passes, followed by standardized high-temperature tempering, were adopted as research objects [38,39]. Comparative UT and SPT tests were performed at room temperature and service elevated temperatures to obtain the engineering stress–strain curves, SPT load–displacement curves and key mechanical characteristic parameters of the two weld metals. Combined with microstructure and fracture morphology observations, the high-temperature deformation and fracture mechanisms were revealed. A performance prediction model was established based on classical UT–SPT correlation equations, and its prediction accuracy was validated to demonstrate the feasibility of SPT in characterizing the high-temperature mechanical properties of P22WM and P91WM. This study provides an experimental basis and methodological support for the in-service performance assessment and residual life prediction of heat-resistant steel welded joints.

## 2. Materials and Methods

The raw materials of P22 and P91 steels are both supplied as pipes with a wall thickness of 50 mm. Two P22 steel pipes were butt girth-welded to prepare P22 butt joints, and two P91 steel pipes were fabricated into P91 butt joints via the same welding process. Before welding, the weld groove was processed in accordance with the dimensions illustrated in Figure 1, and a root gap of 3–5 mm was reserved. Figure 1 presents the schematic diagram of weld groove and welding methods, where (a) shows the groove dimensions and (b) displays the adopted welding procedures. The root passes of both P22 and P91 welded joints were deposited by gas tungsten arc welding (GTAW), and the filling passes were completed by shielded metal arc welding (SMAW) [40,41,42,43]. The detailed welding parameters for P22WM and P91WM are listed in Table 1. For P22WM, CHG-62B3R welding wire (conforming to AWS A5.28, Grade ER90S-B3) was used for GTAW root welding and CHH407R electrode (conforming to AWS A5.5, Grade E9015-B3) for SMAW filling, followed by tempering at 735 ± 15 °C for 2.5 h. For P91WM, CARBOROD CRMO91 welding wire (AWS A5.28, ER90S-B91) was adopted for GTAW and CROMO E91 electrode (AWS A5.5, E9015-B91 H4) for SMAW, with tempering at 750 ± 10 °C for 5 h [44,45]. The heating and cooling rates during tempering treatment were controlled within 125 °C/h for both weld metals. The chemical compositions (wt%) of P22WM and P91WM are provided in Table 2.

For the 50 mm thick weld metal with double-V groove design, multi-pass welding was implemented. A total of 13 welding passes were used for P22WM, including 2 GTAW root passes and 11 SMAW filling and capping passes. For P91WM, 18 welding passes were applied, consisting of 2 GTAW root passes and 16 SMAW filling and capping passes.

Uniform interpass cleaning was performed after each welding pass: weld beads, weld toes and groove sidewalls were thoroughly cleaned using steel wire brushes and grinding wheels to remove welding slag, spatter and oxide scales. All joints were manufactured by single-sided welding with double-sided formation, so back gouging was not required.

During the whole welding procedure, all technological parameters, including current, voltage, travel speed, preheat temperature and interpass temperature were strictly controlled within the standard ranges listed in Table 1. Slight fluctuations of current and voltage inevitably occurred during manual welding, but all values remained within the permitted scope. No excessive parameter deviation or welding defects induced by parameter drift were observed in the finished joints.

Uniaxial tensile (UT) and small punch tensile (SPT) tests were conducted at room temperature (RT) and elevated temperature (ET). The selected room temperature was 25 °C and the selected elevated temperatures were 540 °C for P22WM and 600 °C for P91WM. For SPT tests, all specimens in high temperature were carried out under continuous flowing argon shielding atmosphere to avoid specimen surface oxidation at elevated service temperatures. Figure 2 shows the schematic diagrams of the UT and SPT fixtures. In the SPT fixture (Figure 2b), the lower die diameter a is 4 mm, and the punch ball radius r is 1.25 mm. The disc-shaped SPT specimens are machined to a nominal diameter of 10 mm and an initial thickness t_0_ = 0.5 mm, with specimen surface roughness controlled at *R*_a_ ≤ 0.25 μm. The specific test conditions of UT and SPT, including test temperature and loading rate, are summarized in Table 3. For each test condition, three parallel specimens were fabricated and tested for both uniaxial tensile and small punch tensile tests. Abnormal data from damaged or misaligned specimens were discarded, and the average values of valid data were adopted for further analysis.

The SPT disc specimens and uniaxial tensile specimens were machined entirely from the central region of weld metal, avoiding the fusion line and heat-affected zone; sampling locations and specimen orientation are presented in Figure 3. The loading direction of both specimen types was aligned along the weld bead direction.

Dual axial extensometers with a gauge length of 25 mm were symmetrically mounted parallel to the tensile axis on each specimen; real-time strain was determined by averaging the two measured datasets for both room- and high-temperature uniaxial tensile tests.

Uniaxial tensile tests at room and elevated temperatures were performed in accordance with GB/T 228.1 [46] and GB/T 228.2 [47] respectively; all small punch tensile experiments followed the test specifications of GB/T 29459.2 [48], shown in Table 3.

## 3. Tensile Properties

In this section, uniaxial tensile (UT) and small punch tensile (SPT) tests were performed to systematically characterize the mechanical responses of P22 weld metal (P22WM) and P91 weld metal (P91WM) at room temperature (25 °C) and service elevated temperatures. The engineering stress–strain curves, load–displacement curves, and key mechanical parameters were obtained. The variation laws of strength and plasticity of the two weld metals were compared and analyzed, and the mechanism underlying the property differences was revealed, combined with the microstructures.

Figure 4 presents the engineering stress–strain curves of P22WM and P91WM at room and elevated temperatures. Both materials exhibit typical tensile deformation characteristics of metallic materials. At the initial loading stage, the stress increases linearly with strain, corresponding to the elastic deformation stage. After reaching the yield point, distinct plastic deformation occurs, accompanied by a strain-hardening effect, and the stress rises continuously until the ultimate tensile strength (UTS). Subsequently, necking occurs in the specimen, and the stress drops rapidly until fracture. At room temperature, the strain-hardening stage is more pronounced and the peak stress is higher. At elevated temperatures, the intensified thermal motion of atoms reduces the resistance to dislocation slip, leading to obvious thermal softening and a remarkable decrease in the peak stress. Table 4 lists the key mechanical properties obtained from UT tests, including yield strength (YS), ultimate tensile strength (UTS), and elongation after fracture, which directly reflect the differences in strength and plasticity of the two weld metals at different temperatures.

At room temperature, significant differences exist in the yield strength (YS) and ultimate tensile strength (UTS) between P22WM and P91WM, revealing distinct intrinsic mechanical properties of the two weld metals. Specifically, the YS and UTS of P91WM reach 776 MPa and 866 MPa, respectively, which are much higher than the 543 MPa (YS) and 625 MPa (UTS) of P22WM. Elongation after fracture is a core indicator for characterizing material plasticity. The elongation of P22WM is 18%, significantly higher than the 12% of P91WM, indicating that P22WM possesses superior plastic deformation ability under room-temperature tension, which is consistent with the ductile fracture characteristics observed in the previous fracture analysis.

Under high-temperature conditions, both weld metals undergo thermal softening with a significant degradation in mechanical strength [49]. Among them, the YS and UTS of P22WM decrease to 365 MPa and 382 MPa at 540 °C, respectively, and the elongation after fracture increases to 22%, indicating further optimized high-temperature plasticity. For P91WM, the YS and UTS decrease to 333 MPa and 371 MPa at 600 °C, respectively, with the elongation after fracture rising to 18%. In comparison, P22WM exhibits better high-temperature mechanical stability, while P91WM shows a more significant strength attenuation. Contrary to its performance advantage at room temperature, the YS and UTS of P91WM are both lower than those of P22WM at the corresponding service temperature. In addition, plastic indicators such as elongation after fracture and fracture displacement of P22WM remain superior to those of P91WM at elevated temperatures.

Figure 5 shows the SPT load–displacement curves of P22WM and P91WM at room temperature and elevated temperatures. All curves present a consistent deformation pattern: at the initial loading stage, the load increases linearly with displacement, corresponding to the elastic deformation stage of the material; after reaching the yield characteristic load *F*_e_, the material enters the plastic stage. Once the load reaches the peak load (*F*_m_), the specimen enters the crack propagation stage, the load drops sharply with increasing displacement, and finally, the specimen undergoes through-thickness fracture with the curve flattening out. The peak load at room temperature is higher than that at elevated temperatures for both materials. The load level of P91WM is higher than that of P22WM at room temperature, but slightly lower at service temperature, which is in complete agreement with the strength trend obtained from uniaxial tensile tests [50].

Table 5 lists the characteristic parameters from SPT tests, including the yield characteristic load (*F*_e_) defined by three methods, peak load (*F*_m_), the initial yield force (*F*_i_) adopted in the ALT model, peak displacement (*d*_m_) and fracture displacement (*d*_f_). These parameters can quantitatively describe the strength and plasticity of the weld metals, and all data are supplemented with standard deviation (SD) in the form of mean ± SD.

Overall, the mechanical evolution laws reflected by the SPT characteristic parameters are highly consistent with the uniaxial tensile test results. The yield characteristic load and peak load of P91WM are higher than those of P22WM at room temperature. With increasing temperature, both weld metals undergo thermal softening, and their load levels decrease significantly and approach each other, while P22WM exhibits better high-temperature mechanical stability. In terms of fracture displacement, P22WM outperforms P91WM at both room and elevated temperatures, showing more outstanding plasticity, which is fully consistent with the variation trend of elongation in uniaxial tensile tests.

The SPT peak load can directly reflect the strength level of the material, and the fracture displacement can effectively characterize the plasticity of the material. These two parameters correlate well with the uniaxial tensile mechanical properties, laying a solid data foundation for the subsequent establishment of the UT–SPT property correlation model.

## 4. Microstructure and Fracture Characteristics

### 4.1. Microstructure of BM and WM

Microstructures of BM and WM are shown in Figure 6.

Metallographic specimens were prepared from the base metal (BM) and weld metal (WM) zones of P22 and P91 welded joints. All specimens were mechanically ground sequentially with silicon carbide abrasive papers from 400#, 600#, 800# to 1200#, and then finely polished with diamond suspension on velvet cloth to achieve a mirror-finish surface free of mechanical scratches. For P22 steel (both BM and WM), etching was performed using 4 vol% nital (nitric acid–ethanol) solution. For P91 steel (both BM and WM), a ferric chloride etchant composed of 1 g FeCl_3_, 10 mL concentrated hydrochloric acid and 20 mL deionized water was adopted, with an etching duration of 30 s. The microstructures were characterized and recorded via optical microscopy (OM).

P22 is a 2.25Cr-1Mo ferritic–bainitic heat-resistant steel. The P22 BM possesses a homogeneous microstructure consisting of equiaxed ferrite and granular bainite, with fine Mo- and Cr-rich carbides (M_23_C_6_, Mo_2_C) uniformly dispersed within the matrix. Affected by the rapid solidification of the weld pool and subsequent post-weld tempering, the P22 WM develops a columnar tempered bainitic microstructure; the welding thermal cycle accelerates the precipitation and spheroidization of carbides along grain boundaries, leading to a slightly coarser structure compared with the P22 BM.

The tempered bainite and uniformly dispersed carbides effectively restrict excessive thermal softening at elevated temperatures, which constitutes the essential reason for the better high-temperature mechanical stability and slighter strength attenuation of P22WM, as demonstrated in Section 3. Meanwhile, such a microstructure favors favorable plastic deformation coordination during tensile deformation.

P91 is a modified 9Cr-1Mo tempered martensitic heat-resistant steel. The P91 BM presents a typical tempered martensite lath structure, where prior austenite grain boundaries and martensite lath boundaries are pinned by fine Cr-rich M_23_C_6_ carbides and nanoscale MX (Nb, V) (C, N) precipitates. These nano-precipitates provide strong precipitation strengthening and guarantee the excellent high-temperature creep resistance of P91 BM. In contrast, the P91 WM undergoes rapid melting and solidification during welding, forming a coarse columnar martensitic microstructure. The welding thermal cycle leads to partial dissolution and coarsening of the strengthening precipitates, and the martensitic laths in the WM are less refined than those in the BM, even after post-weld tempering. Benefiting from the standardized welding and high-temperature tempering processes, the P91 WM still presents a relatively uniform microstructure overall. P91 weld metal is dominated by tempered martensite. A large number of Cr-rich carbides precipitate in the weld matrix, which together with Mo and W elements in solid solution contribute to the overall mechanical performance [51].

The dense nano-scaled precipitates impart significant precipitation strengthening to P91WM, leading to its higher room-temperature yield strength and ultimate tensile strength as presented in Section 3. Nevertheless, the coarsening of these strengthening precipitates at high temperatures compromises the strengthening effect, giving rise to more pronounced strength degradation compared with P22WM.

### 4.2. Fracture Morphology

Fracture morphology provides a direct reflection of the fracture behavior and mechanical properties of materials. In this section, the fracture morphologies of uniaxial tensile (UT) and small punch tensile (SPT) specimens of P22 and P91 steel weld metals (WM) were characterized at room and elevated temperatures, and the fracture mechanisms and plasticity differences between the two weld metals were analyzed, combined with their microstructural characteristics. The UT specimens of both materials exhibit obvious ductile fracture features, consisting of a shear lip zone and a fibrous zone. The shear lip zone is relatively smooth, while the fibrous zone is characterized by a large number of dimples.

For the P22 steel weld metal, dense dimples are distributed inside the shear lips (Figure 7a–c). For the elevated-temperature UT fracture (Figure 8a–c), the internal dimples are still densely distributed, indicating that P22WM can still dissipate a large amount of deformation energy through microvoid nucleation, growth and coalescence at high temperatures, showing excellent plasticity and toughness.

For the P91 steel weld metal, obvious macroscopic secondary cracks can be observed on both room-temperature UT fractures (Figure 7d–f) and elevated-temperature UT fractures (Figure 8d–f). With increasing temperature, these secondary cracks become significantly fewer and shallower. These long and deep gullies are radially distributed around the main fracture surface, reflecting the insufficient plastic deformation capacity of P91WM. The inhomogeneous microstructure and hard/brittle phases lead to stress concentration that cannot be released by plastic deformation, inducing crack initiation and propagation, showing a certain brittle tendency [52].

Notably, the same secondary crack features can also be observed on the SPT fractures. The room-temperature SPT fractures (Figure 9a–f) and elevated-temperature SPT fractures (Figure 10a–f) of the two weld metals both present through-thickness fracture characteristics. P22WM SPT fractures are dominated by dense dimples without obvious macroscopic secondary cracks, while P91WM SPT fractures are accompanied by radial secondary cracks with weakened dimple features. Elevated temperature also leads to increased fracture surface roughness and enhanced ductility for both weld metals.

The differences in fracture morphology directly reflect the plasticity levels of the two weld metals. P22WM is typified by spiral shear lips and massive dimples under both UT and SPT conditions at room and elevated temperatures, while P91WM fractures feature a large number of secondary cracks. This further confirms that the plastic deformation coordination of P22WM is superior to that of P91WM, which is consistent with the elongation results shown in Table 4.

## 5. UT–SPT Property Correlation and Model Validation

### 5.1. Relationship Between SPT and UT

The small punch tensile (SPT) curve contains core characteristic parameters such as yield characteristic load *F*_e_, peak load *F*_m_, peak displacement *d*_m_, and fracture displacement *d*_f_, which can be used to inversely derive the yield strength (YS) and ultimate tensile strength (UTS) from uniaxial tensile (UT) tests.

In this study, classical models were adopted to establish a quantitative correlation between the SPT characteristic parameters and UT mechanical properties of P22WM and P91WM. For UTS, two classical correlation models were adopted to establish the linear relationship between SPT characteristic parameters and the UTS obtained from UT tests, namely the classical mechanical properties models (CLA) [31] and the Altstadt model (ALT) [30]; for YS, three yield characteristic loads Fe were obtained by fitting and calculation based on the definitions of Mao [53], CEN [54], and *t*_0_/10.

Linear fitting of the experimental data was conducted based on Equations (1)–(3), and the fitting parameters for different definitions of Fe are listed in Table 6. The fitting results (Figure 11) indicate that significant linear correlations exist between the SPT characteristic parameters and UT mechanical properties for both P22WM and P91WM, with overall high coefficients of determination (*R*^2^). This demonstrates that the established correlation model can accurately reflect the intrinsic mapping relationship between SPT and UT properties of the two weld metals.

(1) $$\sigma_{YS}=\alpha_{1}\frac{F_{e}}{t_{0}^{2}}+\alpha_{2}$$

(2) $$\sigma_{UTS-GAR}=\beta_{1}\frac{F_{m}}{t_{0}\cdot d_{m}}+\beta_{2}$$

(3) $$\sigma_{UTS-ALT}=\beta_{1}\frac{F_{i}}{t_{0}^{2}}+\beta_{2}$$

where *F*_i_ corresponds to the onset of plastic instability in SPT, which is consistent with the necking initiation point in uniaxial tensile tests, thus eliminating the influence of post-necking deformation and crack initiation on the peak load *F*_m_. Specifically, *F*_i_ is defined as the SPT load at a fixed punch displacement *d* = 0.65 mm. This fixed displacement value of *d* = 0.65 mm is proposed in the original literature of this model, which was confirmed via systematic finite element simulation and multi-grade steel experimental verification under the identical SPT geometric configuration (lower die diameter = 4 mm, initial specimen thickness *t*_0_ = 0.5 mm). This position exactly matches the critical moment of plastic instability initiation inside the SPT specimen. If the reference displacement is set below 0.65 mm, the measured load mainly reflects elastic and early strain-hardening response before plastic instability and causes underestimated UTS prediction; when the selected displacement exceeds 0.65 mm, internal necking and microcrack propagation interfere with the load signal, deteriorating the linear correlation accuracy between *F*_i_ and UTS and raising model prediction error. For other key SPT parameters: *F*_e_ is the yield characteristic load obtained from Mao, CEN and *t*_0_/10 criteria; *d*_m_ is the peak displacement corresponding to F_m_; and *d*_f_ represents the fracture displacement of SPT specimens. *α*_1_, *α*_2_, *β*_1_ and *β*_2_ are coefficients determined by fitting the experimental data of this study.

As shown in Table 6, the 95% confidence intervals of all slope parameters (α_1_ for YS models, β_1_ for UTS models) do not include zero, and their corresponding *p*-values are less than 0.05, confirming the statistical significance of the linear correlation between SPT characteristic loads (*F*_e_/*F*_i_) and uniaxial tensile properties (YS/UTS). Although the confidence intervals of some intercepts cross zero, these intercepts are merely mathematical fitting constants with no practical physical meaning, thus not affecting the engineering applicability of the established models.

The YS and UTS were predicted using the above UT–SPT correlation model, and the results are presented in Figure 11. All predicted values stably fall within the preset error bands shown in Figure 12: the predicted YS values are within the 1.15-fold error band, and the prediction accuracy of UTS is better, within the 1.1-fold error band. The overall prediction performance is highly consistent with the research conclusions of base metals. These results confirm that the classical UT–SPT correlation model enables efficient and accurate prediction of the room-temperature and high-temperature mechanical properties of P22WM and P91WM.

### 5.2. Model Accuracy Evaluation

To quantitatively verify the prediction reliability of the established UT-SPT property correlation model, the mean absolute error (MAE), root mean square error (RMSE), and coefficient of determination (*R*^2^) were adopted to conduct quantitative error analysis between the predicted and measured values of yield strength (YS) and ultimate tensile strength (UTS). These error evaluation indicators have been widely used to assess the accuracy of mechanical property prediction models [55,56]. The calculation methods of these indicators are presented in Equations (4)–(6).

(4) $$MAE=\frac{1}{n}\sum_{i=1}^{n}\left|Y_{p}-Y_{e}\right|\;$$

(5) $$RMSE=\sqrt{\frac{1}{n}\sum_{i=1}^{n}{\left(Y_{p}-Y_{e}\right)}^{2}}$$

(6) $$R^{2}=1-\frac{\sum {\left(Y_{p}-Y_{e}\right)}^{2}}{\sum {\left(Y_{p}-Y_{m}\right)}^{2}}$$

The calculated model errors under different definitions of yield characteristic load (*F*_e_) are summarized in Table 7. For yield strength (YS), the Mao model achieves the minimum MAE (5.52) and RMSE (6.765), as well as the highest *R*^2^ (0.999), indicating significantly better prediction accuracy than the CEN and *t*_0_/10 models [29]. The CEN model shows moderate accuracy, while the *t*_0_/10 model presents the largest errors and the poorest fitting performance. For ultimate tensile strength (UTS), the classical CLA model delivers an MAE of 19.415, an RMSE of 22.401, and a determination coefficient *R*^2^ of 0.988. In contrast, the ALT model based on the plastic instability criterion exhibits significantly improved predictive performance, with an MAE of 5.31, an RMSE of 6.603, and an *R*^2^ as high as 0.999. The quantitative comparison confirms that the ALT model is superior to the CLA model for UTS prediction of heat-resistant steel weld metals.

Overall, all UT-SPT correlation models established in this work exhibit prediction accuracy with well-controlled errors, enabling efficient and precise evaluation of the mechanical properties of P22WM and P91WM at both room and elevated temperatures. These results verify the feasibility of the SPT method for evaluating high-temperature mechanical properties of the studied P22 and P91 weld metals under typical service conditions.

It should be noted that the *α* and *β* coefficients obtained in this work are only valid for as-tempered weld metals under short-term monotonic loading. For service-exposed welds with carbide coarsening or creep damage after long-term operation, these correlation coefficients need to be recalibrated. In addition, the linear regression was conducted on four representative working conditions, including two weld metals tested at room temperature and their respective service temperatures. Each test group contained three parallel specimens, and the small standard deviation of test data demonstrates good repeatability. Supported by the results of 95% confidence intervals and *p*-values, the established UT-SPT correlation models are reliable for the investigated materials and typical service scenarios. More test conditions and data points will be supplemented in follow-up studies to improve the universality of the models.

## 6. Conclusions

This study systematically investigated the high-temperature mechanical performance, microstructural evolution and fracture characteristics of P22 and P91 weld metals (P22WM, P91WM) using uniaxial tensile (UT) and small punch tensile (SPT) tests at room temperature and practical service temperatures (540 °C for P22WM, 600 °C for P91WM). The core conclusions are presented as follows:

1. Mechanical properties, microstructure and fracture mechanisms

At room temperature, P91WM possesses a yield strength around 43% higher than P22WM but shows lower ductility. Both materials experience thermal softening under elevated service temperatures. P22WM exhibits outstanding high-temperature structural stability, retaining 24% more strength relative to its room-temperature level than P91WM, and achieves better comprehensive mechanical performance in terms of strength and plasticity at service conditions. Microstructurally, the tempered bainitic matrix with uniformly distributed carbides endows P22WM with strong resistance to high-temperature performance decay. For P91WM, although the tempered martensite with precipitates provides excellent room-temperature strength, the coarsening of strengthening phases at high temperatures leads to substantial strength loss. Fractographic results demonstrate a fully ductile fracture mode for both weld metals. Dense dimples and spiral shear lips are observed on P22WM fracture surfaces, which correspond to superior plastic coordination, while radial secondary cracks on P91WM fractures indicate its relatively poor ductility.

2. Applicability of the SPT technique for weld metal characterization

SPT characteristic parameters, including yield load *F*_e_, peak load *F*_m_ and fracture displacement *d*_f_, show identical variation tendencies with temperature as UT measurements, confirming that SPT can reliably characterize the strength and plasticity of P22WM and P91WM. Compared with conventional UT tests that require large standard specimens, SPT uses miniature disc samples (ø 10 mm × 0.5 mm) and supports minimally destructive sampling within narrow weld regions, making it an ideal technique for performance detection of in-service welded joints. All specimens were machined with loading directions parallel to the weld bead, which effectively mitigates the influence of weld-induced anisotropy and guarantees stable and repeatable test results for as-cast weld microstructures.

3. Prediction accuracy and optimal selection of UT-SPT correlation models

Strong linear correlations exist between SPT characteristic parameters and UT mechanical properties. For yield strength prediction, the Mao model delivers the optimal accuracy with MAE = 5.52, RMSE = 6.765 and R^2^ = 0.999, which is superior to the CEN and *t*_0_/10 models. For ultimate tensile strength prediction, the Altstadt (ALT) model based on the plastic instability criterion outperforms the classical (CLA) model significantly (MAE = 5.31, RMSE = 6.603, R^2^ = 0.999). All predicted results lie within a ±15% error band, proving the robust predictive performance of the developed models for the two weld metals.

4. Engineering implications, limitations and future research

This study has several notable limitations that must be acknowledged before applying the proposed models in engineering practice. The proposed UT-SPT correlation system enables rapid and non-destructive mechanical property evaluation of P22/P91 welded joints under actual service conditions. It is noteworthy that the model coefficients are specifically calibrated for welds produced with the given welding materials, GTAW + SMAW processes and post-weld heat treatment schedules. Recalibration is required before applying these formulas to welds manufactured by different techniques. Furthermore, the current models are applicable only to newly fabricated or short-term serviced weld metals free from carbide coarsening, creep damage and other long-term microstructural degradation. For components operated under prolonged high-temperature exposure, modified models are necessary for accurate residual strength evaluation. Future work will expand the test database by covering more temperature gradients, various welding parameters and artificially aged/creep-damaged specimens, aiming to enhance the general applicability of the correlation models.

## Figures and Tables

**Figure 1:**
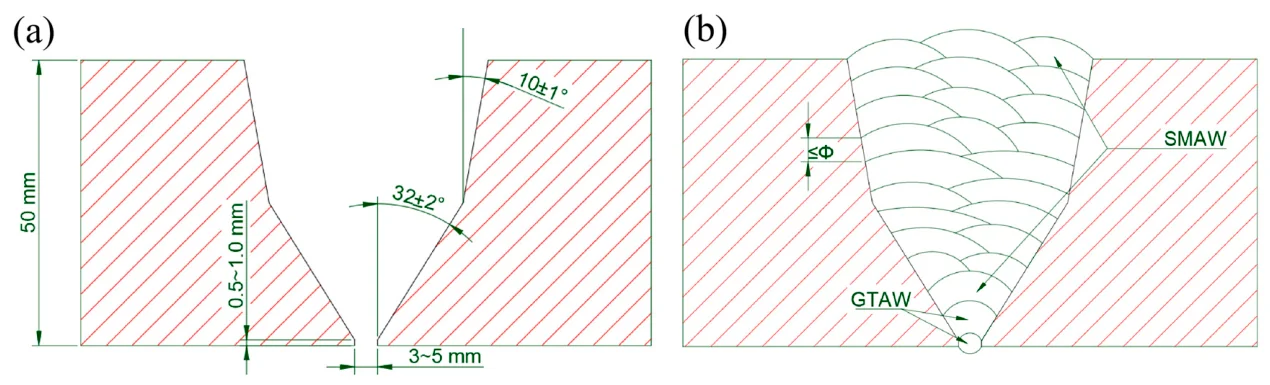
Schematic diagram of weld groove and welding methods: (**a**) groove dimensions; (**b**) welding methods.

**Figure 2:**
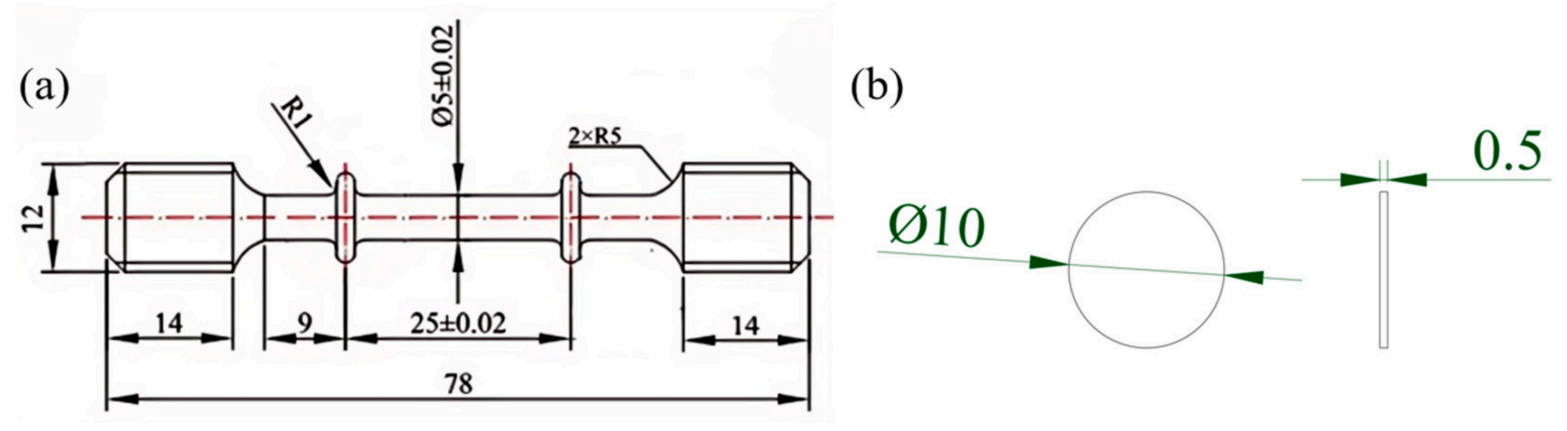
Schematic diagrams of the UT and SPT fixtures: (**a**) UT; (**b**) SPT.

**Figure 3:**
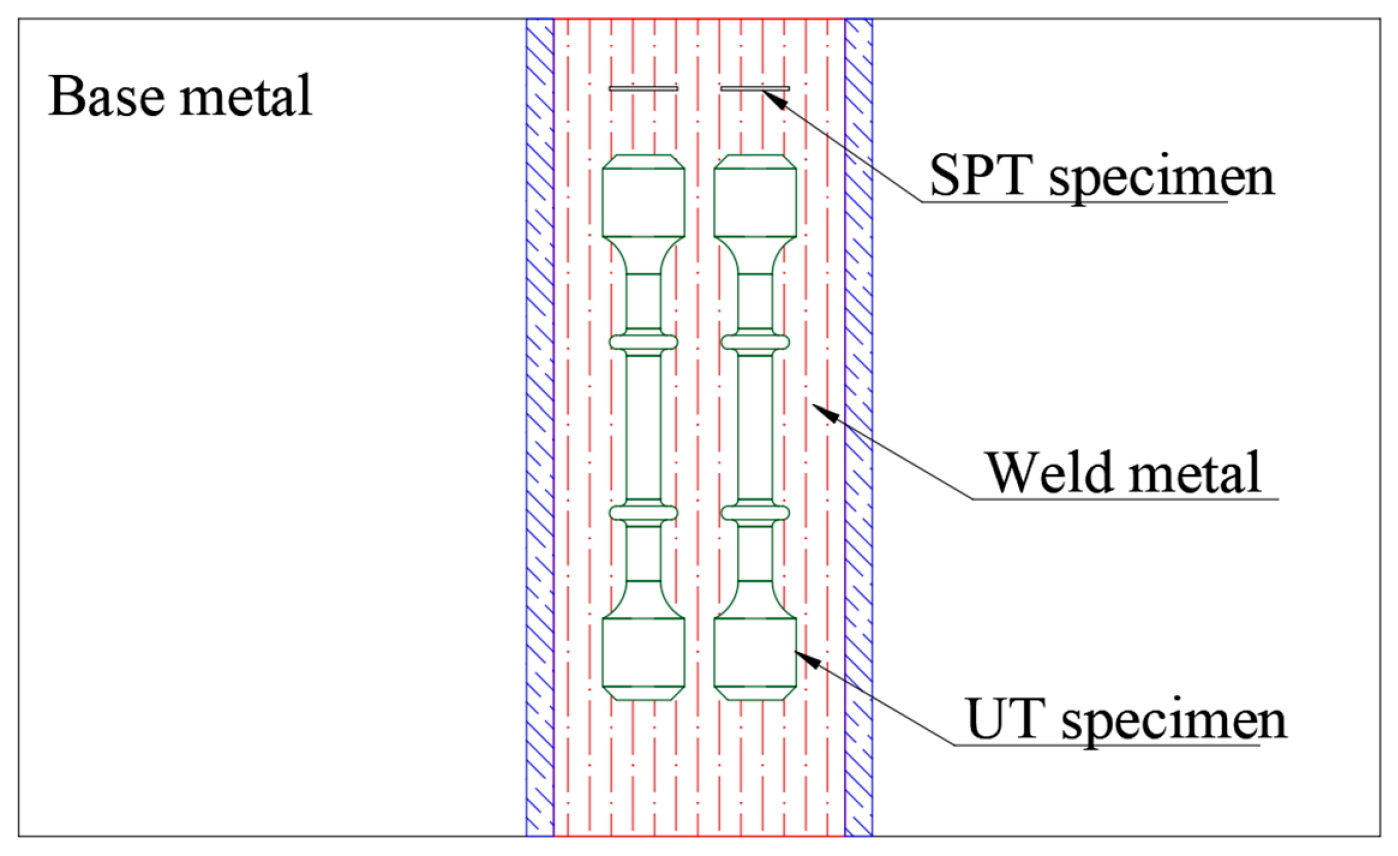
Sampling locations of uniaxial tensile and small punch specimens.

**Figure 4:**
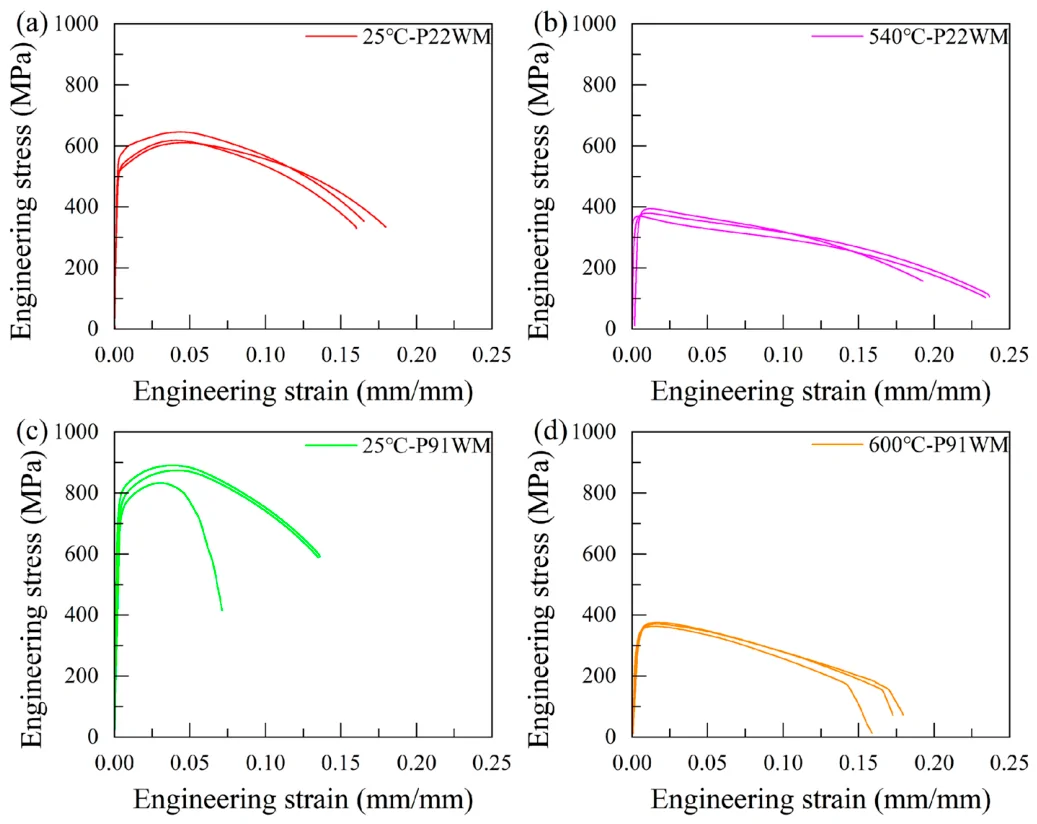
Engineering strain and engineering stress curve of P22WM and P91WM: (**a**) 25 °C-P22WM, (**b**) 540 °C-P22WM, (**c**) 25 °C-P91WM, (**d**) 600 °C-P91WM.

**Figure 5:**
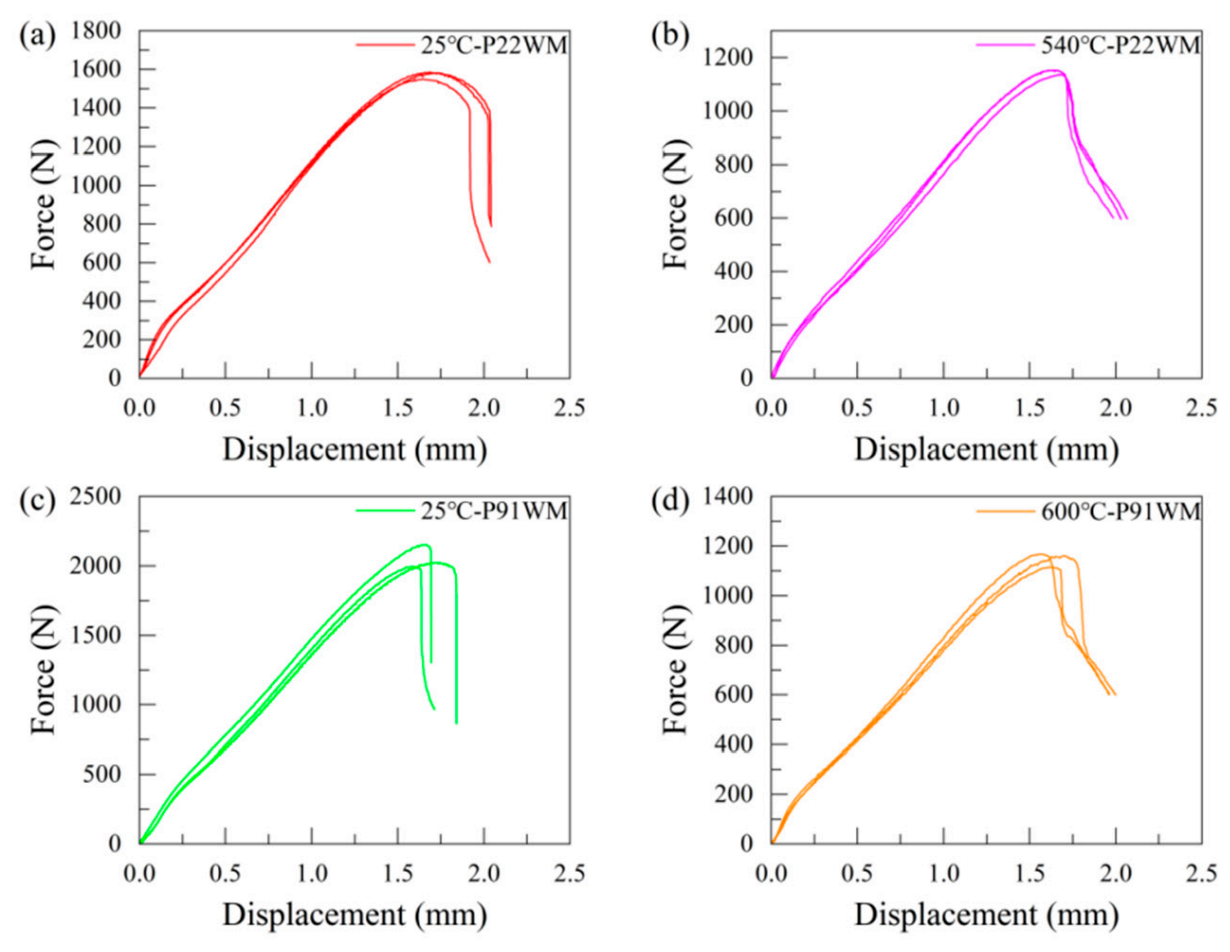
SPT curves of P22WM and P91WM: (**a**) 25 °C-P22WM, (**b**) 540 °C-P22WM, (**c**) 25 °C-P91WM, (**d**) 600 °C-P91WM.

**Figure 6:**
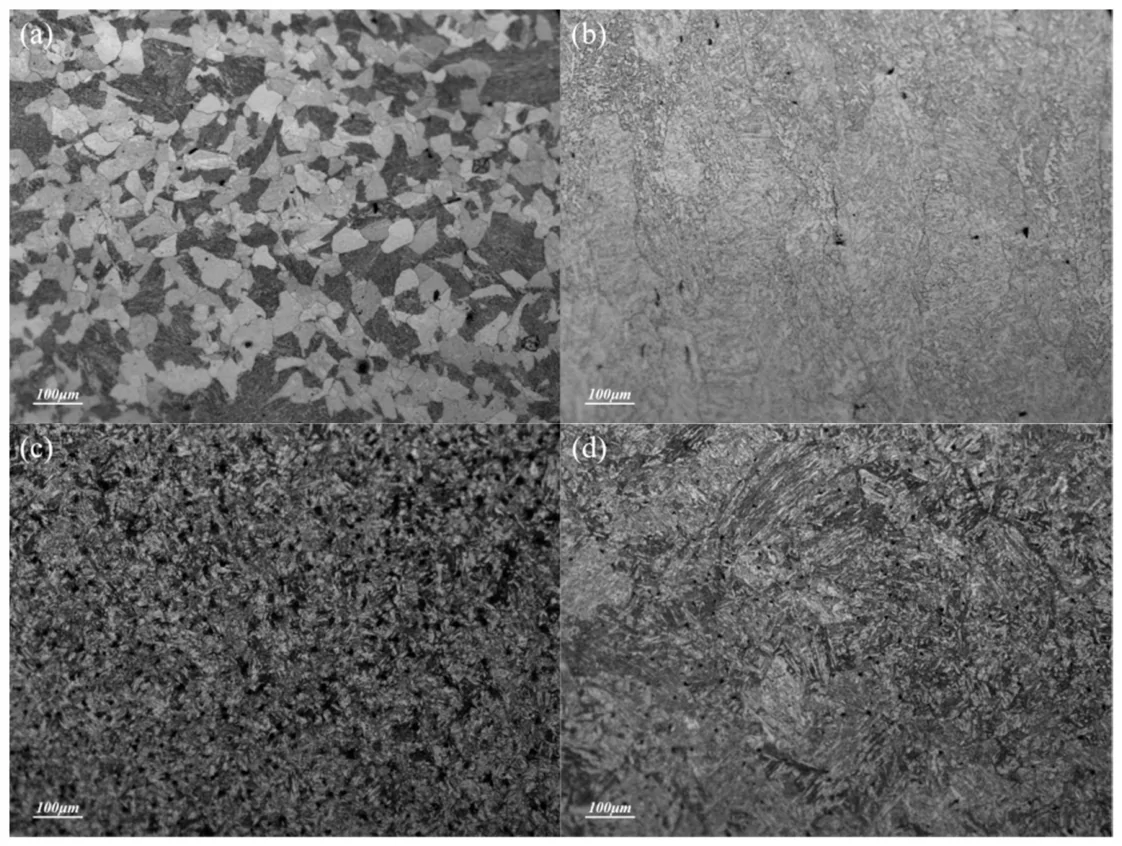
Optical micrographs of: (**a**) P22 BM, (**b**) P22 WM, (**c**) P91 BM, (**d**) P91 WM.

**Figure 7:**
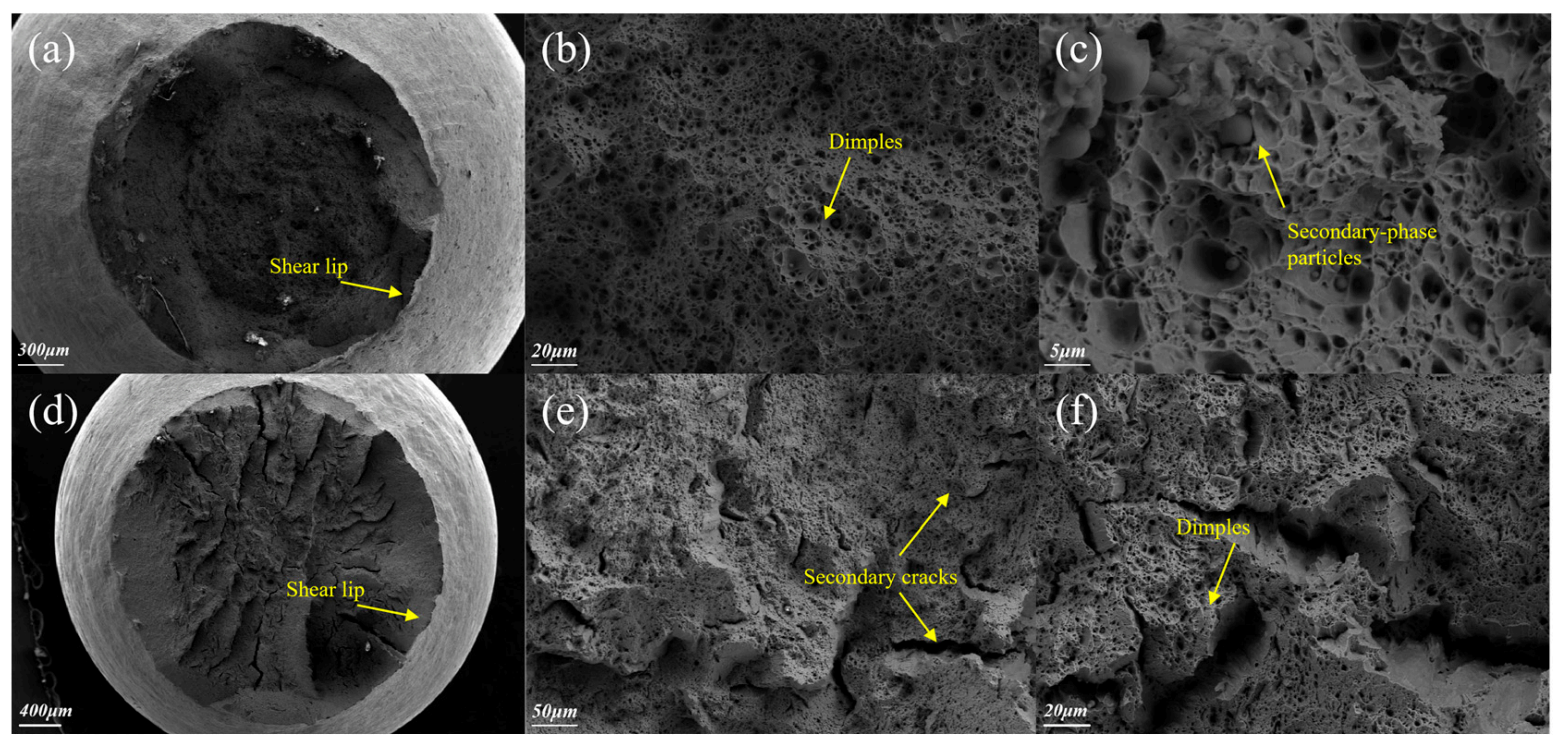
SEM fractographs of UT specimens at room temperature: (**a**–**c**) P22WM; (**d**–**f**) P91WM.

**Figure 8:**
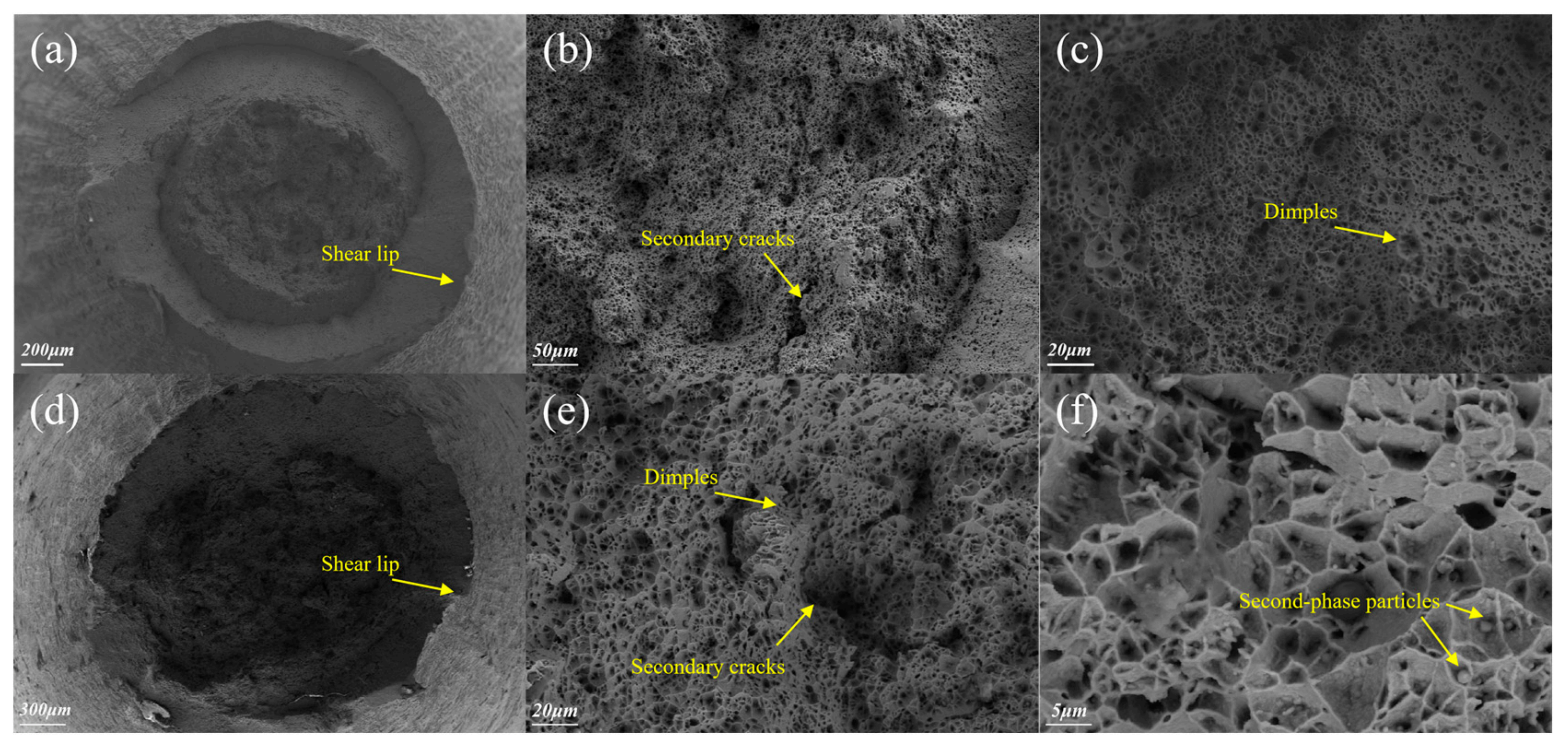
SEM fractographs of UT specimens at elevated temperature: (**a**–**c**): P22WM, (**d**–**f**): P91WM.

**Figure 9:**
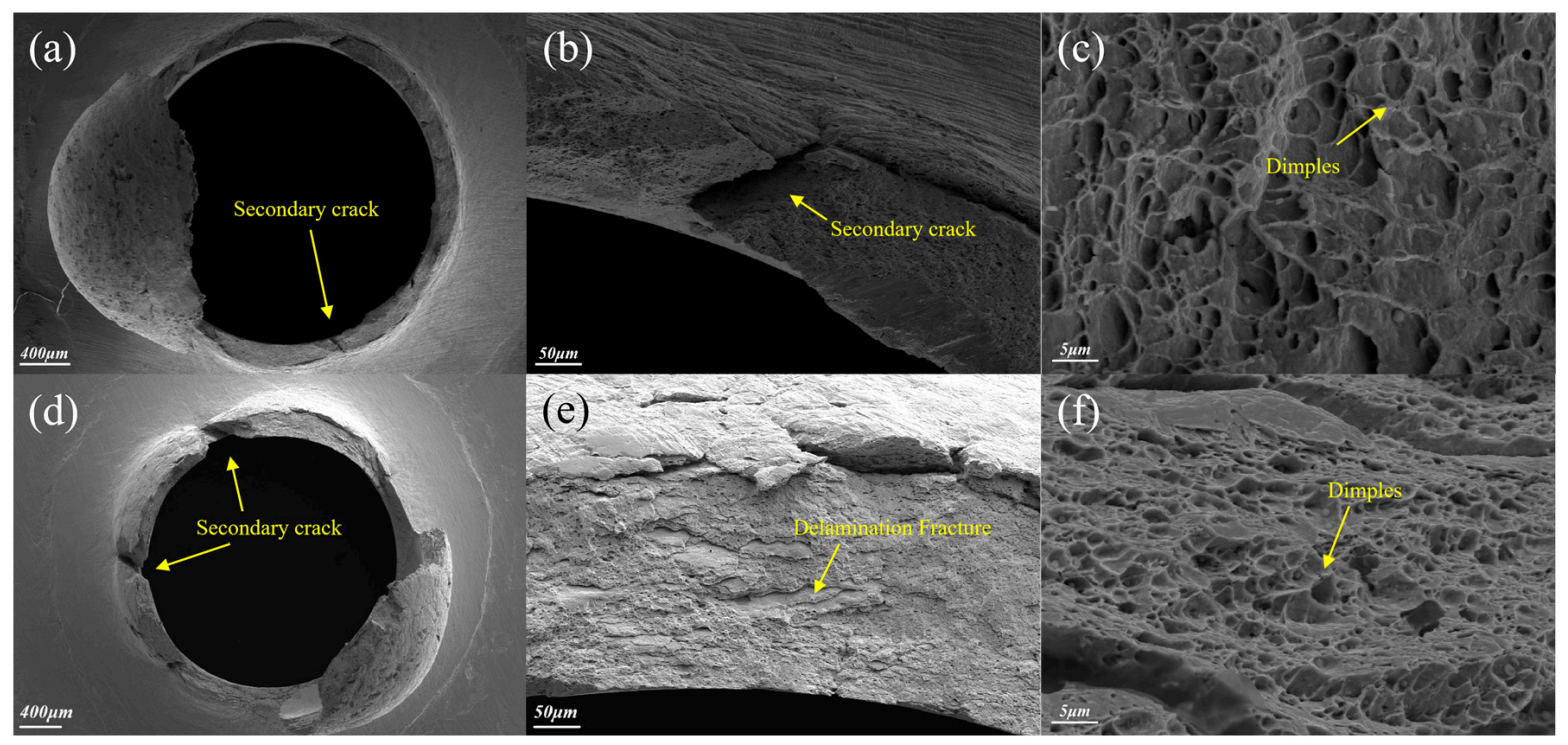
SEM fractographs of SPT specimens at room temperature: (**a**–**c**): P22WM, (**d**–**f**): P91WM.

**Figure 10:**
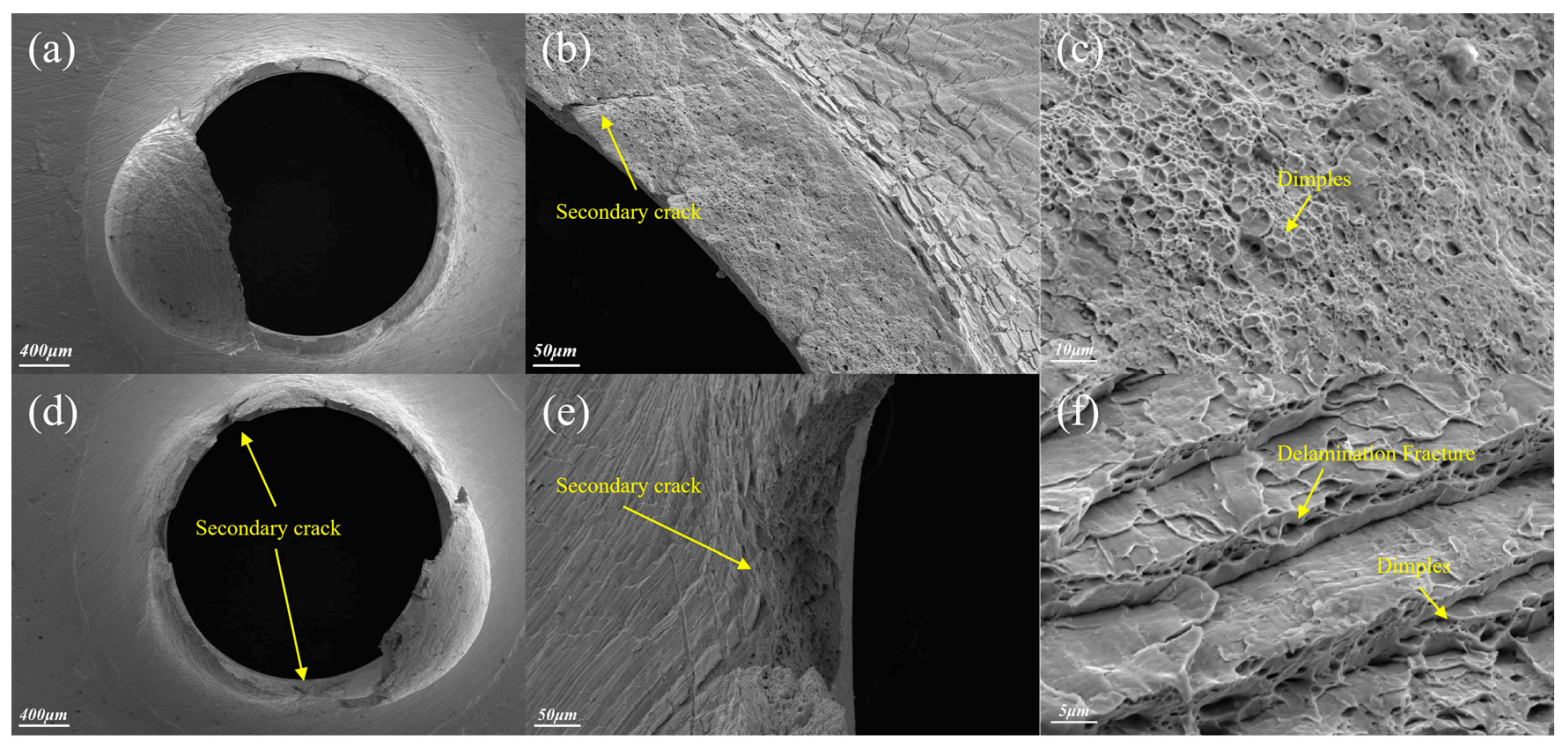
SEM fractographs of SPT specimens at elevated temperature: (**a**–**c**): P22WM, (**d**–**f**): P91WM.

**Figure 11:**
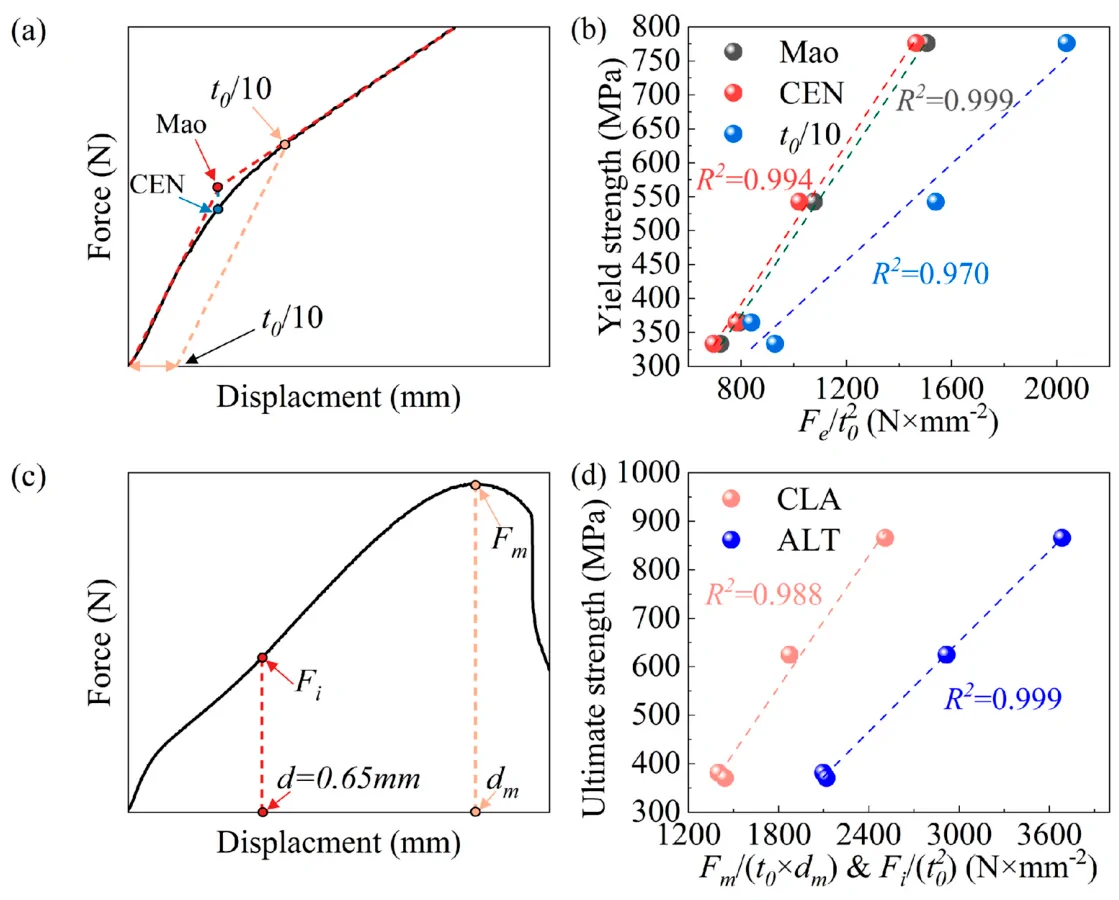
SPT fitting results. (**a**) Defining of *F*_e_, (**b**) YS fitting results, (**c**) defining of *F*_i_ and *F*_m_, (**d**) YS fitting results.

**Figure 12:**
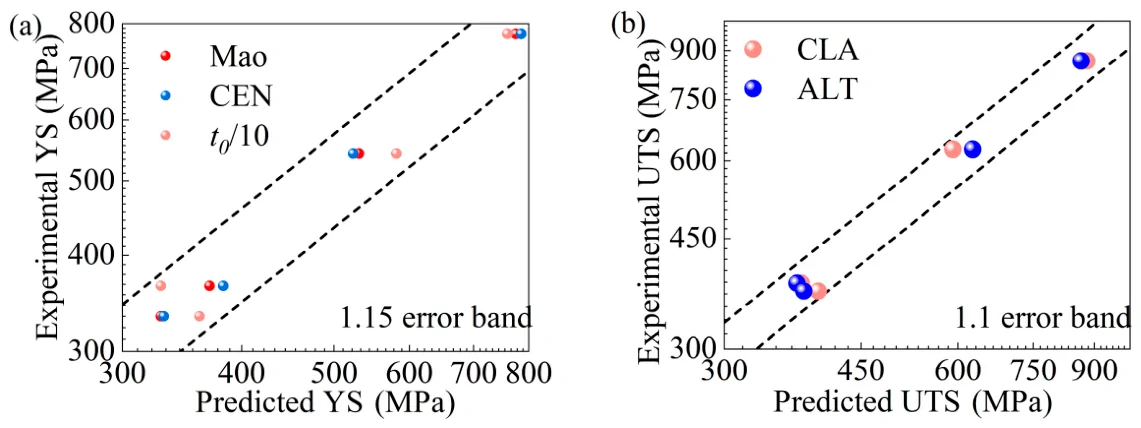
SPT predicted results. (**a**) YS, (**b**) UTS.

**Table 1:** Welding methods for P22 and P91 steel welds.

Item	P22WM	P91WM
Welding method	GTAW root + SMAW filling	GTAW root + SMAW filling
Welding position	6G (45° fixed welding)	6G (45° fixed welding)
Preheating temperature (°C)	GTAW: 150–300; SMAW: 200–300	GTAW: 130–250; SMAW: 180–250
Interpass temperature (°C)	200–300	180–250
Welding current (A)	GTAW: 100–140; SMAW: 100–160	GTAW: 100–130; SMAW: 100–135
Welding voltage (V)	GTAW: 10–14; SMAW: 20–25	GTAW: 10–14; SMAW: 20–25
Tempering temperature (°C)	735 ± 15	750 ± 10
Tempering holding time	2.5 h	5 h
Heating/cooling rate (°C/h)	≤125	≤125

**Table 2:** Chemical composition of P22WM and P91WM (wt%).

Material	C	Si	Mn	Cr	Mo	V	Nb	Ni	Al	Fe
P22WM	0.09	0.52	0.51	2.34	1.24	—	—	0.04	0.02	Bat.
P91WM	0.13	0.34	0.98	9.55	1.08	0.20	0.05	0.04	0.01	Bat.

**Table 3:** Tensile test condition of UT and SPT.

Material	UT		SPT	
	Test Temperature (°C)	Test Rate (mm/min)	Test Temperature (°C)	Test Rate (mm/min)
P22WM	25	0.375	25	0.2
	540	0.105	540	0.2
P91WM	25	0.375	25	0.2
	600	0.105	600	0.2

**Table 4:** UT properties’ results and standard deviation of P22WM and P91WM.

Material	Temperature (°C)	YS (MPa)	UTS (MPa)	Elongation (%)
P22WM	25	543 ± 28	625 ± 18	18 ± 2
	540	365 ± 13	382 ± 13	22 ± 2
P91WM	25	776 ± 30	866 ± 30	12 ± 3
	600	333 ± 10	371 ± 6	18 ± 2

Note: All data are presented as mean ± standard deviation (SD), *n* = 3.

**Table 5:** SPT characteristic parameters’ results and standard deviation of P22WM and P91WM.

Material	Temperature (°C)	*F*_e_ (N)			*F*_m_ (N)	*F*_i_ (N)	*d*_m_ (mm)	*d*_f_ (mm)
		Mao	CEN	*t*_0_/10				
P22WM	25	269 ± 21	255 ± 27	385 ± 66	1573 ± 20	729 ± 28	1.68 ± 0.03	1.99 ± 0.06
	540	199 ± 7	196 ± 3	210 ± 39	1156 ± 9	524 ± 23	1.65 ± 0.02	1.74 ± 0.02
P91WM	25	376 ± 24	366 ± 17	509 ± 102	2058 ± 38	921 ± 88	1.64 ± 0.06	1.72 ± 0.10
	600	181 ± 14	174 ± 17	232 ± 17	1169 ± 9	530 ± 20	1.62 ± 0.08	1.74 ± 0.07

Note: All data are presented as mean ± standard deviation (SD), *n* = 3.

**Table 6:** Fitting parameters and 95% confidence intervals of Equations (1)–(3).

	*α*_1_	Confidence Interval	*α*_2_	Confidence Interval		*β*_1_	Confidence Interval	*β*_2_	Confidence Interval
Mao	0.57	[0.51, 0.63]	−82.68	[−147, −18]	CLA	0.45	[0.30, 0.61]	−258.02	[−543, 27]
CEN	0.59	[0.45, 0.73]	−78.94	[−224, 66]					
					ALT	0.31	[0.28, 0.34]	−277.68	[−363, −192]
*t*_0_/10	0.36	[0.17, 0.55]	26.75	[−247, 300]					

**Table 7:** MAE RMSE and *R*^2^ calculation results for mechanical properties.

Properties	Methods	MAE	RMSE	*R* ^2^
YS	Mao	5.52	6.765	0.999
	CEN	11.94	13.863	0.994
	*t*_0_/10	29.71	30.901	0.969
UTS	CLA	19.415	22.401	0.988
	ALT	5.31	6.603	0.999

## Data Availability

The data presented in this study are available on request from the corresponding author. The data are not publicly available due to privacy.
